# Semi-automation of process analytics reduces operator effect

**DOI:** 10.1007/s00449-019-02254-y

**Published:** 2019-12-07

**Authors:** A. Christler, E. Felföldi, M. Mosor, D. Sauer, N. Walch, A. Dürauer, A. Jungbauer

**Affiliations:** 1 Austrian Centre of Industrial Biotechnology, Muthgasse 11, 1190 Vienna, Austria; 2 Institute of Bioprocess Science and Engineering, Department of Biotechnology, University of Natural Resources and Life Sciences Vienna, Muthgasse 18, 1190 Vienna, Austria

**Keywords:** Liquid handling, Pipetting, PicoGreen, Endotoxin, Host cell proteins, dsDNA, ELISA

## Abstract

The aim of this study was to semi-automate process analytics for the quantification of common impurities in downstream processing such as host cell DNA, host cell proteins and endotoxins using a commercial liquid handling station. By semi-automation, the work load to fully analyze the elution peak of a purification run was reduced by at least 2.41 h. The relative standard deviation of results among different operators over a time span of up to 6 months was at the best reduced by half, e.g. from 13.7 to 7.1% in dsDNA analysis. Automation did not improve the reproducibility of results produced by one operator but released time for data evaluation and interpretation or planning of experiments. Overall, semi-automation of process analytics reduced operator-specific influence on test results. Such robust and reproducible analytics is fundamental to establish process analytical technology and get downstream processing ready for Quality by Design approaches.

**Electronic supplementary material:**

The online version of this article (10.1007/s00449-019-02254-y) contains supplementary material, which is available to authorized users.

## Introduction

Semi-automation is the compromise to accelerate process development while maintaining high precision and reasonable costs in an environment where the number of samples is manageable. The benefits of full automation are often overestimated. Full automation is desirable if highest productivity is anticipated and to eliminate most human influence. Since the beginning of the 1990s, especially after the turn of the millennium, automated liquid handling systems enabled high-throughput methods and thus revolutionized laboratory work. This is highlighted by numbers of publications in different fields of application such as nucleic acid synthesis and analysis, protein refolding, production host clone screening, process development, diagnostics, cell culture and others [1-9]. However, setting up fully automated methods is laborious, time-consuming [10, 11] and investment costs for fully automated equipment are high. This implies a long-term commitment for a specific assay. Usually 3–5 years is necessary for full automation to depreciate the high upfront costs for the equipment. Chan emphasizes that high-volume testing, meaning many samples, may benefit using semi-automated steps for the most labor-intensive steps [12].

Conventional as well as DoE-based process development, process modelling, and manufacturing processes of recombinant proteins require in-process analytics to monitor the quality of the product and depletion of impurities. Especially, biopharmaceuticals are subjected to stringent regulatory requirements. Appropriate analytical methods must provide information about the content of target protein, its activity and show that critical impurities such as DNA, host cell proteins (HCP), endotoxins and product-related impurities are cleared from the final protein product below certain levels as specified by national and international authorities. Accuracy and precision of analytical results are usually assured by method qualification, validation and continuous training of operators. However, Pandya et al. found that their long-term stability testing of protein therapeutics was obscured by the systematic differences in manual pipetting between operators [13], commonly known as “operator effect”. Moreover, manual pipetting is highly repetitive and might lead to fatigue and possibly repetitive strain injury [14]. Therefore, we hypothesize that an appropriate automation strategy will improve reproducibility of results in a long-term study involving multiple operators, increase security of data delivery due to an operator-independent analytical workflow and protect staff from adverse effects of monotonous work. Manpower released by automating repetitive work can be deployed for more demanding tasks and thus increase productivity.

In the present literature, either very specialized (semi-automated) applications were reported or liquid handling stations (LHS) with high capacities and functionalities were used. We extend the field by describing semi-automated methods for four different common biochemical assays (dsDNA, HCP, endotoxins, binding affinity) using a simple commercial LHS and comparing them to manual assay performance. The adaptation of the analytical protocols to the space and tools of the LHS is described. Spectroscopically detectable model substances were used to discriminate the influence of sample dilution steps only to deviations obtained for the individual assays. The outcome of the semi-automated protocols was compared to conventional manual analytics in terms of time efficiency, precision and reproducibility. Semi-automation of analytics reduced long-term variability of analytical results and improved the confidence in the subsequent data application.

## Materials and methods

### Instrumentation

A liquid handling station epMotion®5073 (Eppendorf, Germany) equipped with a thermal module (0–110 °C), a single-channel liquid transfer tool (40–1000 µL) and an 8-channel tool (20–300 µL) was used. Plates were incubated in a Thermomixer Comfort MTP (Eppendorf, Germany). Spectroscopic measurements were performed using a plate reader Infinite M200 PRO (TECAN, USA) with a dual pump dispense unit. Preparative chromatography was carried out on an ÄKTA pure 25 workstation (GE Healthcare, USA).

### Chemicals

Chemicals were purchased from E. Merck (Germany) in analytical grade unless specified differently. Green fluorescent protein (GFP) was produced in-house and kindly provided by Prof. Rainer Hahn.

### Protein samples

An IgG1 monoclonal antibody (mAb) was produced in CHO cell culture, harvested, and captured by Protein A affinity chromatography as described in [15]. IgG1 concentrations were determined by high-performance monolith affinity chromatography as described in [16]. Human fibroblast growth factor 2 (FGF-2) was expressed in *E. coli*, captured by cation exchange or affinity chromatography, polished by hydrophobic interaction chromatography, and quantified by reversed phase HPLC, all as described in [17].

### Biochemical assays

The principles described below are valid for both manual and semi-automated procedures. Serial 1:2 dilutions were done in 350 µL NUNC® 96F 96-well microplates (Thermo Fisher Scientific, USA). From the dilution plates, 100 µL were transferred to the measurement plates (Corning® Costar 350 µl 96-well plates, Sigma-Aldrich/Merck, USA) or MaxiSorp™ Immuno ELISA plates (Thermo Fisher Scientific, USA). Data were evaluated using MS Excel (Microsoft, USA). In semi-automated methods, samples for binding affinity and endotoxins were pre-diluted in 96-well 2 mL deepwell® plates (VWR, USA). In manual methods, the dilution was carried out in 1.5 mL (Sarstedt, Germany) and 2 mL reaction tubes (Eppendorf, Germany).

#### Host cell protein (HCP) ELISA

Capture and detection antibodies and HCP standards were purchased from Cygnus, USA. Product numbers are given in brackets. ELISA plates were coated with 0.25 µg of anti-*E. coli* HCP (AP117) or 0.5 µg anti-CHO HCP (3G-0016-AF) antibody per well in 100 µL of 0.2 M sodium carbonate buffer (pH 9.3–9.5) for 2 h at 37 °C/350 rpm. Plates were washed three times with 300 µL of PBS (137 mM NaCl, 2.7 mM KCl, 10 mM Na_2_HPO_4_, 1.8 mM KH_2_PO_4_) containing 0.05% Tween 20 (pH 7.2–7.6) per well. Plates were blocked with 300 µL 3% BSA in PBS per well overnight at 4 °C. The blocked plates were washed as before. Samples and concentrated *E. coli* or CHO HCP antigen (F413H or F553H) were diluted in sample buffer (1% BSA, 0.05% Tween 20 in PBS) and incubated for 1 h at 37 °C/350 rpm. Plates were washed as before and incubated with 100 µL/well of a 0.5 µg/mL (0.05 µg/well) detection antibody solution (anti-*E. coli*-HCP, F411C or anti-CHO–HCP, F551C) conjugated with horseradish peroxidase (HRP) in sample buffer for 1 h at 37 °C/350 rpm. Plates were washed again as before and incubated with 100 µL/well of a tetramethylbenzidine (TMB) substrate (Bio-Rad, USA) for 30 min at room temperature without shaking. The HRP reaction was stopped by adding 50 µL/well of 1 N sulfuric acid. Absorbance was measured at 450 nm and at 630 nm as reference which was subtracted from the absorbance at 450 nm. Average blank was subtracted from all measurements. A quadratic calibration curve was fitted through the standard measurements. The calibration range for *E. coli* HCP was 0.39–25 ng/mL and 2.11–135 ng/mL for CHO HCP.

#### Double-stranded (ds) DNA quantification by Quant-iT™ PicoGreen® assay

DsDNA concentrations were determined with Quant-iT™ PicoGreen® assay (Invitrogen, USA). 20 × TE buffer was diluted 1:20 with RO-water to a working concentration of 10 mM Tris–HCl, 1 mM EDTA, pH 7.5 (1 × TE). Samples and *λ* DNA standard were diluted in 1 × TE. 100 µL of Quant-iT™ PicoGreen® working solution in 1 × TE was added to each well. After incubation for 2 min at room temperature in the dark, fluorescence was measured using an excitation wavelength of 480 nm and emission wavelength of 520 nm (filter with a bandwidth of ± 20 nm). Average blank was subtracted from all measurements. A linear calibration curve was fitted through the standard measurements and the origin of the coordinate system (0,0). The calibration range for *E. coli* DNA was 3.91–500 ng/mL and 1.95–250 ng/mL for CHO DNA.

#### Endotoxin quantification with recombinant factor C-based assay

Endotoxins were determined using EndoZyme® II recombinant Factor C (rFC)-based assay kit (Hyglos, Germany). Samples and standards were diluted in endotoxin-free water. Vigorous mixing (30–120 s on orbital shaker at 1400 rpm or ten cycles of aspiration and dispense at a speed of 11 mm/s) was applied to disperse the analytes homogeneously. The plate was heated to 37 °C. 100 µL of enzyme–substrate solution was added to each sample and standard dilution. Signal intensities were measured at an excitation wavelength of 380 nm and emission wavelength of 445 nm. Plates were incubated at 37 °C for 75 min without shaking. Signals at time 0 were subtracted from signals after 75 min. Average blank was subtracted from all measurements. A linear calibration curve was fitted through the standard measurements and the origin of the coordinate system (0,0). The calibration range was 0.01–5 Endotoxin Units (EU)/mL.

#### Determination of ligand binding affinity with a surface plasmon resonance (SPR)-based assay

Binding affinities of anti-TNFα-IgG against TNFα (10,602-HNAE-100, Sino Biological, China) and of FGF-2 to FGF-receptor 2 were determined by a SPR assay using a Biacore 2000 system (GE Healthcare, USA) as described in [17].

#### Quality criteria

For the standard curve fit of PicoGreen®, HCP ELISA and endotoxin assays, a value of the determination coefficient *R*^2^ of at least 0.999 was accepted. A maximum tolerable deviation from the nominal concentration (bias) of ± 15% was allowed. For each reported target response, at least three consecutive values from different dilutions were averaged that give a coefficient of variation (CV) ≤ 20%. The lower limit of detection (LLOD) was calculated as the average of at least three blank measurements plus three times the standard deviation of blanks. The lower limit of quantification (LLOQ) was calculated as average blank plus ten times the standard deviation of blanks. The upper limit of quantification (ULOQ) was the highest calibration standard.

### Comparison of accuracy and precision of automated dilutions compared to manual

To compare the processes of sample and standard dilution only, model substances were used that can be detected spectroscopically. Solutions of myoglobin, the pH indicator bromocresol purple, and green fluorescent protein (GFP) were prepared in the respective buffer system. Dilutions of model substance solutions were treated like process samples to compare the manual and semi-automated methods as close to real situations as possible. Random concentration levels were assumed for the model solutions and dilutions calculated accordingly. Averages of 2 replicates for each dilution level were compared and the respective differences between manual and automated results plotted over the average absorbance value according to Bland and Altman, 1999 [18].

## Results

### Transfer of analytical methods from manual to semi-automated processes

**Fig. 1:**
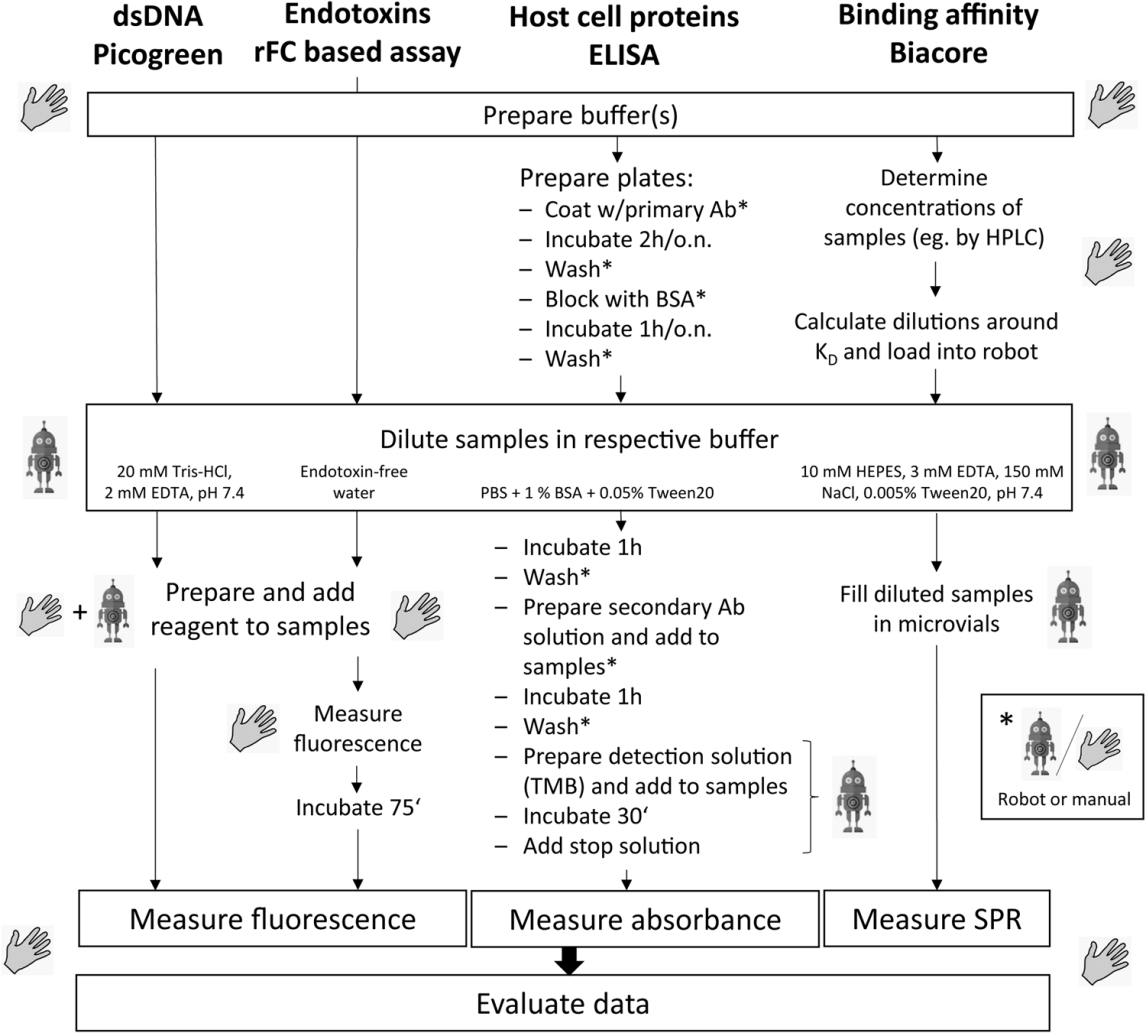
Steps in the four semi-automated analytical procedures. Hands symbolize manual actions, robots denote automated steps. Asterisks denote steps with an automated option. Abbreviations: *rFC* recombinant factor C, *HCP* host cell proteins, *TMB* tetramethylbenzidin, *Ab* antibody, *HPLC* high-pressure liquid chromatography, *SPR* surface plasmon resonance

Four analytical methods commonly applied for process analytics in downstream processing of recombinant proteins were semi-automated using a commercial LHS. The analytical methods comprised the quantification of host cell dsDNA, HCP, bacterial endotoxins and binding assays for potency estimation. The methods were adjusted for processes capturing an IgG1 antibody and a basic fibroblast growth factor-2 (FGF-2), respectively. Different steps of the analytical procedures were automated (Fig. 1): sample dilution, filling of diluted samples in vials (binding affinity), and addition of reagents to samples (dsDNA and HCP). Sample dilution was identified as highly potential for automation, since this step consumed the most operator time and was very repetitive. For example, in the early-stage purification, endotoxin concentrations in samples exceeded the ULOQ up to 37,600-fold (Tables 1 and 2). Even samples with analyte concentrations within the quantification ranges usually must be diluted to eliminate or reduce matrix influence which otherwise can impair accurate quantification. This was the case for DNA determination in samples of IgG1 where the measured concentrations never exceeded the ULOQ.

**Table 1:** Ranges of analytes in process samples from capture stage purification, analytical ranges and analyte concentrations relative to upper limits of quantification (ULOQ)

Protein sample	Component	Concentration range in fractions (mL^−1^)	Analytical range of assay (mL^−1^)	Analyte concentrations in % of ULOQ
FGF-2	Product	0.01–42 mg		
	DNA	50–2000 ng	3.91–500 ng	10–400%
	HCP	20–500 ng	0.39–25 ng	80–2000%
	Endotoxins	20–188,000 EU	0.01–5 EU	400–3760 000%
IgG	Product	0.004–32 mg		
	DNA	≤ 1.95*–100 ng	1.95–250 ng	≤ 40%
	HCP	0.060–30 µg	2.11–135 ng	44–22,222%

^*^Below lower limit of quantification (LLOQ)

**Table 2:** Ranges of analytes in process samples after FGF-2 polishing, analytical range of endotoxin assay and endotoxin concentrations relative to upper limit of quantification (ULOQ)

Protein sample	Component	Concentration range in fractions (mL^−1^)	Analytical range of assay (mL^−1^)	Analyte concentrations in % of ULOQ
FGF-2	Product	0.07–7.3 mg		
	DNA	< LLOQ		
	HCP	< LLOQ		
	Endotoxins	20–7000 EU	0.01–5 EU	400–140,000%

Differences between manual and semi-automated pipetting mainly concerned (1) the available space in the LHS, (2) the number and volume ranges of the pipetting tools and (3) the operating direction of the multichannel tool. In most of the methods, more units of labware (plates, reservoir holder, tube racks, tips) were needed compared to the available space on worktable of the LHS (Fig. S1 in Supplementary Material). In these cases, labware had to be interchanged manually during a method. The number of exchanges could be reduced using deepwell plates for sample dilution instead of reaction tubes. This allowed to divide dilution factors into smaller steps or to process more samples at a time.

#### Host cell dsDNA

DNA staining by Quant-iT™ PicoGreen® is a fast and sensitive method for dsDNA quantification. Automation of this assay was straightforward due to its short protocol and convenient pipetting behavior of the dilution buffer (low viscosity, high surface tension). Aliquot dispense of DNA staining reagent was semi-automated using a dispense unit attached to the spectroscopic plate reader to reduce light-induced degradation of the dye and exposure time of the operators to the hazardous reagent. The minimum dilution for all samples was 1:2.

#### Protein-specific binding affinity

To determine the binding affinity by SPR as a measure for the product´s potency, the protein concentration must be reduced to levels around the expected dissociation constant (*K*_D_). Since the process samples contained different initial product concentrations (Tables 1 and 2), they must be normalized to the same concentration in the beginning of sample dilution. In the semi-automated procedure, this was achieved by manually generating csv-files based on templates and importing the volumetric information contained in them into the LHS, similar to the way described in [19]. Samples were filled in the dilution plate manually and all further dilution steps were executed automatically. Templates were created to calculate volumes to be used by the LHS (see Fig. S2A in Supplementary Material). This allowed fast and flexible sample processing in routine analytics.

To avoid disturbance of SPR measurements by gas bubbles, the running buffer was degassed by ultrasonication prior to sample dilution. Dissolution of air during sample dilution and filling as well as trapping of air bubbles in sample vials must be avoided. This was achieved by adjusting the speed of dispense to 3–4 mm/s. No higher frequency of gas bubble disturbance was observed in the sensorgrams of samples diluted automatically compared to manual sample dilution (data not shown). Therefore, we conclude that the robotic mixing by repeated aspiration and dispense did not dissolve more air than manual mixing on an orbital shaker.

**Fig. 2:**
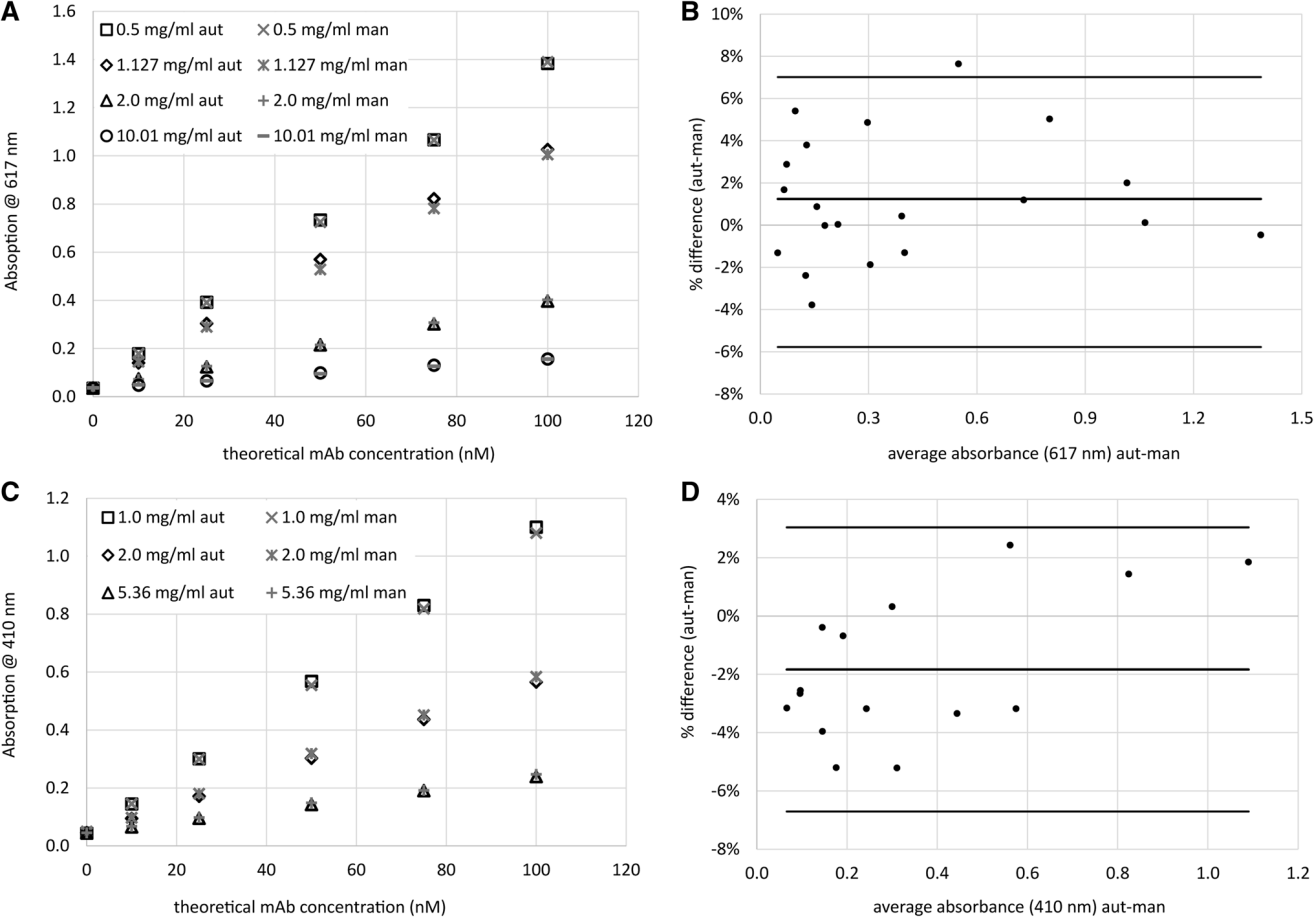
Absorbance measurements of model substances bromocresol purple (**a**–**b**) and myoglobin (**c**–**d**) assuming different initial mAb concentrations. **b** and **d** show relative differences over the concentration ranges of the prepared samples with averages and limits of agreement

First experiments indicated that measurement of SPR response and fitting of the data to a binding model caused more variance in the final analytical result than the sample dilution process. Therefore, model substances were used to assess the differences between manual and semi-automated sample dilution for binding affinity measurements. Buffered solutions of bromocresol purple and myoglobin were diluted in accordance to both protocols, manual and semi-automated. After normalization to a common concentration representing 100 nM antibody concentration, five consecutive independent dilutions with dilution factors between 1.3 and 10 were prepared. Limits of agreement defined as the 95% confidence intervals [18] were − 5.8% and + 7.0% for bromocresol purple (Fig. 2a, b) and − 6.7% and + 3% for myoglobin (Fig. 2c, d). The differences of the bromocresol purple sample contents were evenly distributed around zero. For the myoglobin solution, differences for smaller concentrations (higher dilution factors) were mostly negative and positive for the higher concentrations. Average differences of semi-automated results compared to manual dilution were + 1.2% and − 1.8% for bromocresol purple and myoglobin, respectively. These are acceptable ranges for use in the assay.

#### HCP ELISA

Immunological methods such as western blot and ELISA are the standard for HCP detection and quantification. HCP ELISA was the longest and most challenging protocol to semi-automate. One reason was that the buffers used contained BSA and Tween 20 to reduce unspecific binding of matrix components. Detergents facilitate air bubble formation in pipette tips upon aspiration and foaming on liquid surfaces upon dispense through a reduced surface tension and increased viscosity of the solution. Air bubbles and foam can lead to volume inaccuracies and must be avoided. On the other hand, pipetting speed must be high enough to ensure complete mixing. In our case, this was achieved by repeated (3–7x) aspiration and dispense at a speed of 1 mm/s. Exact values might vary with equipment.

**Fig. 3:**
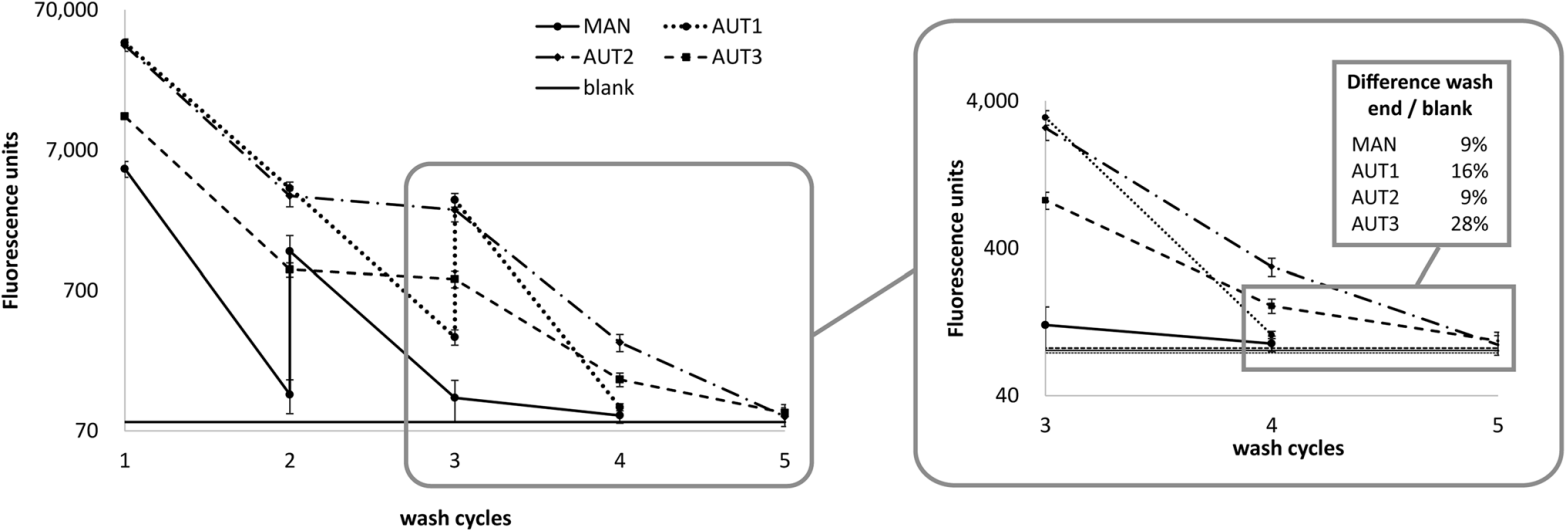
Wash-out of GFP by manual and different semi-automated procedures monitored by fluorescence detection. Left panel: absolute fluorescence counts after each wash cycle. Signal amplification factor was increased when the signal dropped below 700 FU to distinguish signal from blank. Right panel: final fluorescence signals compared to blank (solid line ± one standard deviation)

A plate wash procedure involving four cycles of aspiration and dispense of wash solution was developed on the LHS. Equivalence of wash efficiency to manual wash was shown using GFP solution and measuring fluorescence after each wash cycle (Fig. 3). Three slightly different automated protocols were compared to manual: AUT1 and AUT2 include manual emptying of residual liquid in the plate after the wash procedure of three or four wash cycles, respectively. For the third protocol (AUT3), manual emptying of residual liquid was done prior to four wash cycles. The residual fluorescence determined after the entire protocol was used to measure the efficiency of the procedures. Using AUT1 and AUT2, a depletion in the range of the manual protocol was achieved. AUT3 showed a 20% higher residual fluorescence than the manual protocol. AUT2 plate wash procedure was applied for HCP ELISA assays and compared to the manual protocol. Both plate wash methods produced equivalent results (data not shown), thus the semi-automated plate wash procedure with four wash cycles with manual emptying of residual liquid in the end was chosen as standard protocol.

#### Endotoxin

Efficient mixing is critical during sample preparation for endotoxin detection due to the amphiphilic nature of the lipopolysaccharides. According to the kit manufacturer, samples and standard solutions should be shaken for 1–2 min at 1400 rpm on an orbital shaker. Aspirating and dispensing solutions in the LHS 10 × at a speed of 9–14 mm/s resulted in equivalent signals of standard dilutions compared to manual mixing and dilution (data not shown). The manufacturer moreover recommended using glass containers rather than polymeric due to a potentially higher surface adherence of lipopolysaccharides to polymeric material [20, 21]. Recovery of endotoxins from glass and polymeric containers was tested by comparing signals of dilutions performed in the respective containers. Equivalent signals were obtained even at low concentrations (0.01–0.1 EU/mL). Full analyte recovery from polymeric containers enabled the dilution of samples and standard in polystyrene deepwell plates instead of in glass vials. Since in the early stage purification of *E.coli* homogenates, endotoxin levels were very high (up to 188 000 EU/ml), the number of vials required for dilution would have exceeded the available space in the LHS. Thus, the applicability of multiwell plates was an important prerequisite to semi-automate this assay.

#### Labware compatibility between manual and semi-automated methods

Most of the standard labware used in manual methods can be also used in the LHS as they are stored in a built-in database. Special pipette tips, reservoirs, reservoir holder and a tube rack were purchased with the instrument. Accurate information about geometries of the used labware were required for exact liquid aspiration and dispense. Starting liquid levels of buffers and samples were detected by the optical sensor of the LHS. The rise and fall of filling levels upon aspiration and dispense was then calculated by the system using the container geometry. Thus, a strongly deviating liquid level, due to for example deviating container geometry, can lead to distorted aspiration or dispense and thus produce wrong results. Therefore, specification of the labware is crucial for correct pipetting. Special labware can be sent to the LHS manufacturer to establish a dataset and use it in the LHS.

#### Pipetting tools

Plunger-operated pipettes usually achieve greatest accuracy and precision at the upper limit of their volume ranges. The accuracy of the single-channel tool was checked gravimetrically and spectroscopically. At the minimum volume recommended by the manufacturer for the single channel tool (40 µL), with ELISA sample buffer which is the fluid with the highest viscosity and the lowest surface tension of the solutions tested and considered similar to complex biological samples from early-stage purification (compare Sect. 3.1.1), the relative error was between + 3.3 and + 4.1% (*n* = 6). A minimal working volume for sample aspiration with the single-channel tool was set from experience at a value of 75 µL. For accurate serial dilutions, the 8-channel tool’s precision was tested. At the upper limit (300 µL), an error of + 1.1 ± 0.8% (*n* = 4) was observed for pipetting of water and reuse of tips. This bias was reduced to − 0.4 ± 0.3% (*n* = 4) when fresh tips were used after each cycle which is in the specification range given by the vendor.

**Fig. 4:**
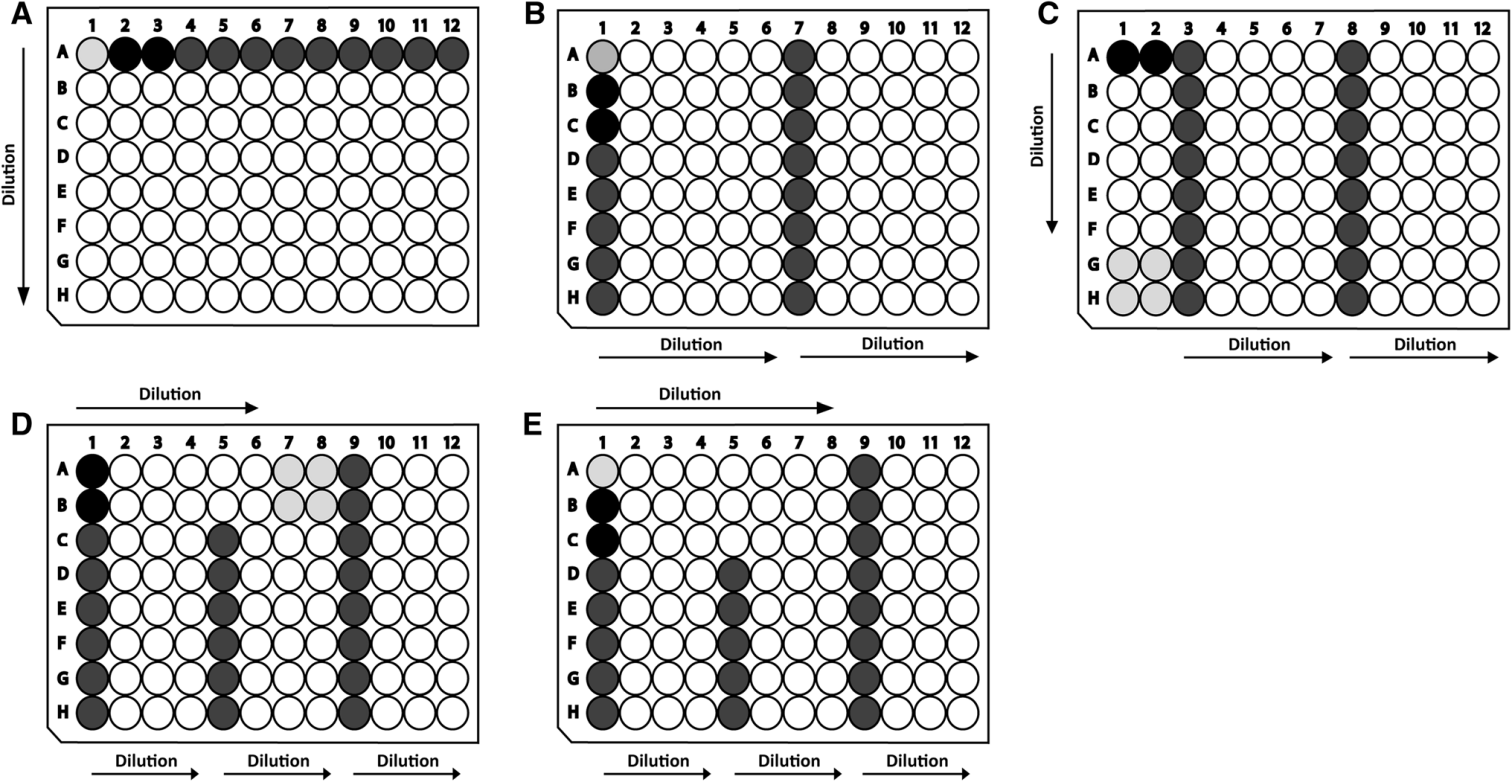
Arrangements of samples on 96-well dilution plates used for different assays. Blank, reference standards and samples are placed in wells indicated in light, dark and medium grey, respectively, and diluted serially 1:2 in the direction of the arrows. **a** Application scheme in all manual assays. Automated assays: **b** ELISA, **c** endotoxins (capture), **d** endotoxins (polishing), **e** dsDNA

In the manual protocols, 12 specimens (samples, standard, blank, references) were diluted with a 12-channel pipette in vertical direction on dilution plates (Fig. 4a). In automated liquid handling systems, multichannel tools usually have eight channels and they are operated in horizontal direction. Various positionings of samples on dilution plates were applied to meet the needs of the different assays (Fig. 4b–e). Two parameters determined the most suitable type of arrangement: (1) the range of analytes in samples and (2) the analytical range and linearity of the assays. Assays with a large linear range, such as the PicoGreen® DNA assay and the recombinant Factor C-based endotoxin assay, required a lower number of measurements (e.g. four) to achieve results with sufficient accuracy. Nevertheless, due to the large range of endotoxin concentrations over the elution peak, five serial dilution levels per sample were used. After another purification step (FGF-2 polishing), less dilution levels were needed due to a more narrow range of concentrations in samples. The range of DNA levels in all samples was small enough so that four 1:2 serial dilutions per sample were sufficient. If the assay response increases non-linearly with analyte concentration or only in a small range as in the case of HCP ELISA, more dilutions are preferable. Due to the very high HCP concentrations compared to the analytical range and the assay non-linearity, six dilutions per sample were used. Schemes in Fig. 4c–e allowed to measure all fractions of a run on one plate. For HCP ELISA, 1.5 plates were required to analyze all fractions of one chromatographic run.

### Performance comparison of semi-automated and manual procedures

#### Time

**Fig. 5:**
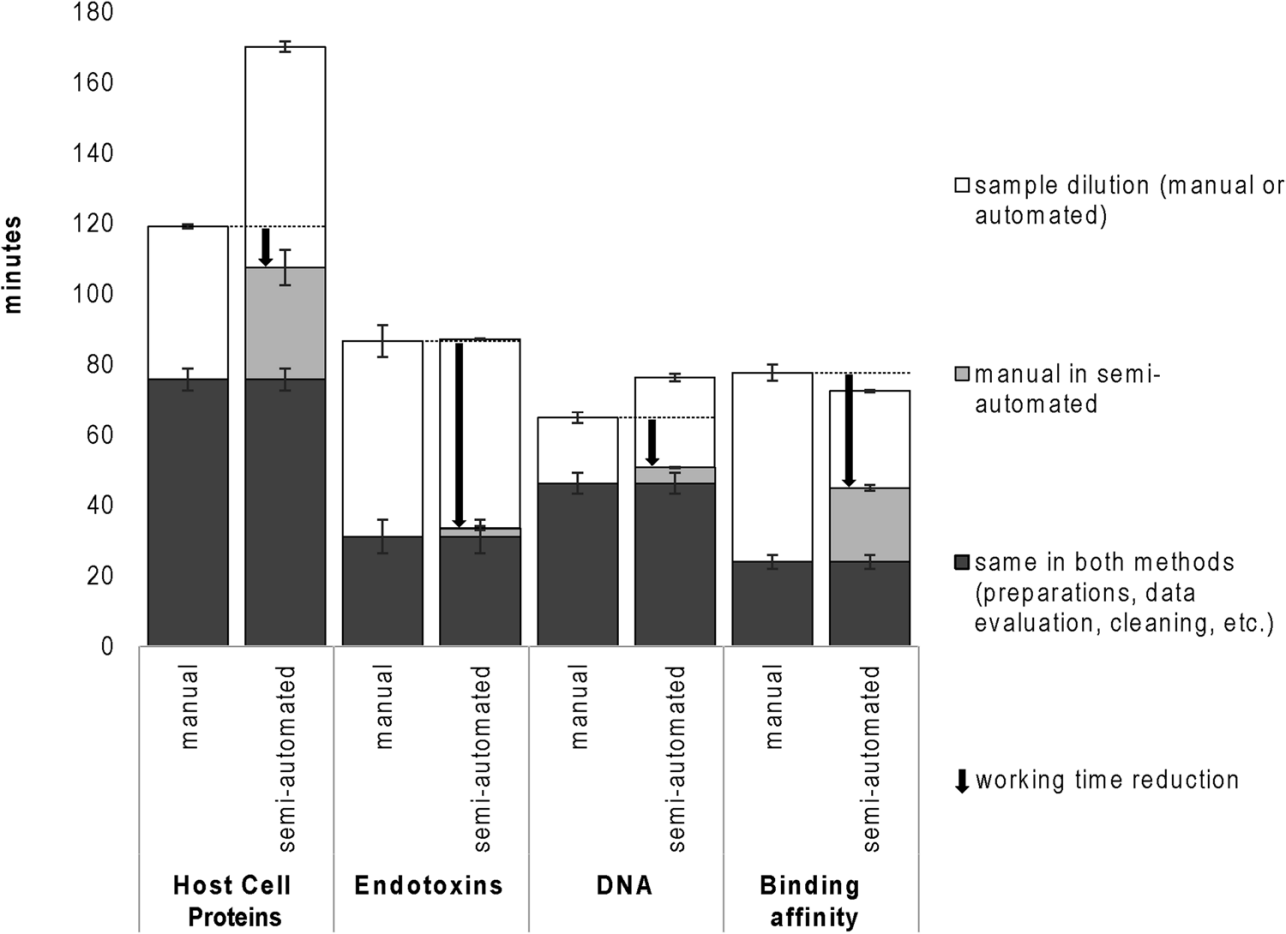
Total and operator working times for typical sets of samples (all 15–20 fractions of one purification run for HCP, DNA and endotoxins, eight fractions for binding affinity). Error bars represent ± 1 standard deviation of three or four measurements. Dark grey parts are performed manually

In a preparative chromatographic purification run in lab scale, typically 15–20 fractions are collected during elution which are then analyzed. In our assays for DNA, HCP and endotoxins, all samples were analyzed together, while for binding affinity determination, two times eight samples were determined consecutively, because many dilution steps were required. In Fig. 5, the total times of manual and semi-automated methods are compared for a polishing run. Details are given in the Supplementary Material, Table S1. The total times per assay were almost equal for manual and semi-automated methods with the exception of ELISA, where the semi-automated procedure requires relatively much operator time. Additionally, the speed of pipetting must be low in this assay due to the buffers’ tendency to form air bubbles during pipetting.

The working time reduction by semi-automation of each assay was calculated as the difference between the sum of the manual steps in semi-automated method and the total time needed for manual execution. With the semi-automated procedures, operator working time could be reduced on average by 11.6–53.2 min per analysis. In total, for the analysis of product purity and potency, about 225 min or 3.75 h of sample dilution could be eliminated by semi-automation and around 144 min or 2.41 h of working time saved. The time saving will be even higher for earlier purification steps (product capture) where impurity levels are higher.

#### Accuracy and reproducibility

**Fig. 6:**
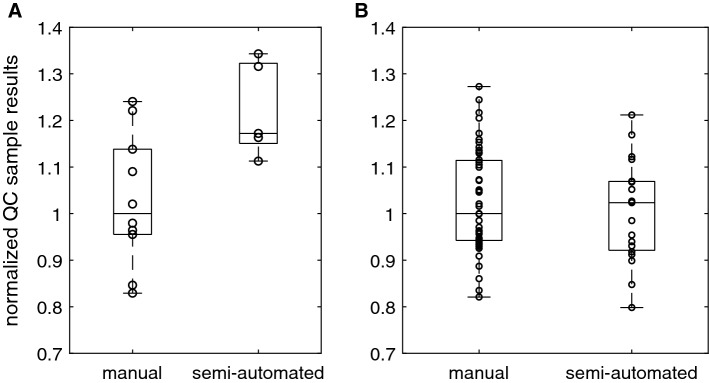
Reproducibility in (**a**) PicoGreen DNA and (**b**) ELISA HCP assays

The inter-assay variations of DNA and HCP measurements were estimated using a quality control (QC) sample in each assay. Comparison of results from manual and semi-automated methods was sensible only for fully quantitative methods. Due to low DNA concentrations close to the LLOQ in IgG1 samples, matrix effects were dominant resulting in poor dilutional linearity. Poor dilutional linearity was also observed in HCP measurements of FGF-2 samples. We, therefore, considered these results as semi-quantitative and did not use them for accuracy and precision assessments. Measurements of the QC samples were normalized by division by the median of the respective manual assay results which were 76.29 µg/ml for DNA and 1.195 µg/ml for CHO HCP. The median of the semi-automated DNA measurements was 17% higher than the manual assay results while the distribution was narrower (Fig. 6a). For CHO HCP ELISA, a slightly larger inter-assay variability (10.9%) was found compared to manual (8.3%). The difference of average results was − 2% (Fig. 6b). Only one operator was involved producing the data, whereas for the DNA assay four operators were involved.

## Discussion

Quality testing of products is a bottleneck in biopharmaceutical process development and manufacturing with its stringent regulatory requirements. Specifically, the laborious and time-consuming repetitive sample dilution are driving forces for the implementation of automated equipment. In this work, biochemical analytical protocols for the analysis of samples from chromatographic protein purification steps were semi-automated to reduce operator hands-on-time, parallelize workflows and accelerate development projects which depend on analytical results. Semi-automated protocols were developed starting from manual procedures and adjusted for typical protein samples from different expression hosts and purification processes, containing large ranges of analyte concentrations. Equivalent results were obtained compared to established manual methods except for DNA measurements, where higher values were obtained. The use of model substances allowed to directly compare the dilution processes and exclude variance added by the detection method. This approach was most useful in methods using measurements of SPR or enzyme activity because these steps caused more variance to the result than the sample dilution process. Enzyme activity depends on temperature which is not perfectly distributed in 96-well plates and in thermoshakers [22] and might influence the final results.

Operator working times were reduced to very different extents for the different assays by the semi-automated procedures. The largest benefits in this respect were observed for binding affinity and endotoxin determinations, because large dilution factors and rigorous mixing was required.

ELISA plate wash was mostly done manually, because the automated plate wash took around 6.7 times as long as manual plate wash (around 40 min for 2 plates compared to around 6 min manually). Since the automated and the manual protocols led to results of same accuracy and reproducibility, plate wash was assigned optionally automated or manual in the semi-automated procedure depending on the individual time schedule and preference of the operator. Similarly, reagent dispense was left to the operators’ discretion to carry out manually or semi-automated, since the methods were equal in terms of time and quality. Automation of reagent dispense with the liquid handling station was, however, beneficial in routine analytics when several steps could be combined such as staining, incubation and stop in ELISA (Fig. 1). Also, dispensing light-sensitive reagents such as PicoGreen® automatically in the plate reader reduces light-induced degradation and thus increases sensitivity compared to manual dispense. The reduced handling with the potentially mutagenic reagent moreover improved working safety.

Semi-automation lead to a reduction of operator hands-on-time and influence, but it required adjustments to overcome technical limitations of the LHS such as restricted space and pipetting tools. Generally, larger volumes were used compared to manual to increase precision. Compatibility of the LHS with common laboratory containers and plates is advantageous since it reduces dependency on any special materials and products from a designated vendor. Deepwell plates allowed to perform more sample dilutions on the same footprint compared to using reaction tubes. Thereby, the number of manual exchanges during a method could be reduced. More efficient sample patterns on multiwell plates could be carried out with the LHS. Thereby, the required quantities of reagents and materials as well as effort for data evaluation were reduced. Extensive repetitive movements as required for operating plunger-driven manual pipettes and thus the risk of repetitive strain injuries in the hands, arms and shoulders [14, 23] were reduced by automation.

The design of the control unit user interface was found to be important for user efficiency and comfort. The software must not only be functional and enable flexibility, but also has to be designed in a way allowing intuitive use by scientists and all staff not trained in automation. The interface must be easy to understand considering and using common laboratory workstyles such as sequences in multiwell plates. With the liquid handling system used in this study, automation was most beneficial for routine analyses. Adaptation of semi-automated methods to higher or lower analyte concentrations was time-consuming. Extensive operator training was necessary.

The different reproducibility observed for DNA quantification compared to HCP content estimation (Fig. 6) indicate that the remaining manual liquid transfer steps, e.g. of the assay calibration standard, still impact the final results. Systematic differences between the pipetting techniques of operators were reported to cause significant deviations in results [1]. A dilution-dependent positive bias at low dilution factors (up to 1:10) and a negative bias of samples diluted more than 1:100 was reported [13] and also observed in our data (Fig. S4). This might be one reason for the difference of average manual and semi-automated results for DNA measurements. In our case, reduction of manual pipetting in the assay protocol reduced the inter-assay variation of DNA measurements to about half. Thus, reproducibility between operators was improved which is essential if data are generated over longer periods of time by several people. The CHO host cell protein ELISA data showed that semi-automation did not improve precision and reproducibility of one trained operator.

We suggest to implement the proposed methodology in early development where screening of different product variants (lead candidates), materials and/or process conditions is required. Semi-automation allows more flexibility required in this stage of development compared to full automation.

We see potential of semi-automation for any other (spectroscopic) analytical method that requires several sample dilutions and/or aliquot reagent dispense after a defined incubation time, such as UV/Vis absorption- or fluorescence-based quantifications, total protein determination (e.g. Bradford staining), nanoparticle tracking (e.g. NanoSight), or viral titer quantification (e.g. TCID50), to name some examples.

## Conclusion and outlook

In the presented study, semi-automation of sample preparation for biochemical analyses reduced operator times and operator-specific influence, thereby increased data consistency, and unburdened staff from repetitive physical tasks such as sample dilution. Critical issues of typical analytical methods for quality testing such as HCP ELISA, DNA quantification, enzymatic endotoxin detection and SPR-based binding assay were discussed. We showed which challenges might arise in common analytical procedures regarding semi-automation with a simple liquid handling station and how these challenges can be addressed.

For commissioners of analytics, automation bears the potential to reduce personnel cost and on the other hand, reduce variability due to operator influence. Less extensive training of staff might be necessary since the critical steps of sample and standard dilution are carried out by a robot. Procedures should be easily adaptable and (re-)validated. For the concerned staff, automation reduces the risk of hand and shoulder ailments.

A next step to further improve analytical workflows and data quality will be assay miniaturization to reduce sample and reagent volumes, material costs, time and potentially accuracy and reproducibility [24].

To get biopharmaceutical ready for Quality-by-Design approaches as recommended by the authorities, such robust and reproducible analytics can be used to calibrate process analytical technology (PAT) and model-based control algorithms.

## Electronic supplementary material

Below is the link to the electronic supplementary material.

Supplementary file1 (PDF 382 kb)
