# Supplementary material for: Semi-automation of process analytics reduces operator effect
Source: Bioprocess Biosyst Eng. 2019 Dec 7;43(5):753–64. doi: 10.1007/s00449-019-02254-y (PMC7125066; doi:10.1007/s00449-019-02254-y)
Supplement: Supplementary file 1 — Supplementary file1 (PDF 382 kb) [file 449_2019_2254_MOESM1_ESM.pdf]

Supplementary Material

Bioprocess and Biosystems Engineering, Springer

Semi-automation of process analytics reduces operator effect

A. Christler[1], E. Felföldi[1], M. Mosor[1], D. Sauer[1], N. Walch[1], A. Dürauer[1,2], A. Jungbauer[1,2]

[1] Austrian Centre of Industrial Biotechnology, Muthgasse 11, A-1190 Vienna, Austria
[2] Institute of Bioprocess Science and Engineering, Department of Biotechnology, University of Natural Resources and Life Sciences, Vienna, Muthgasse 18, A-1190 Vienna, Austria
E-mail: alois.jungbauer@boku.ac.at

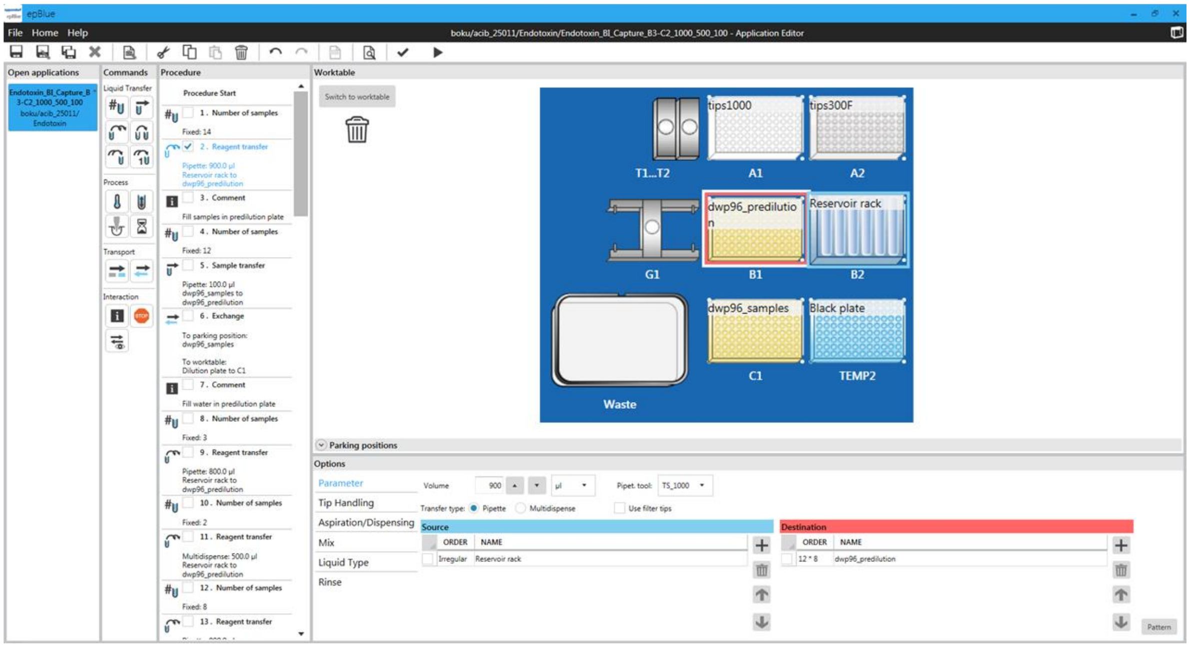

Figure S1: Representation of the epBlue^{TM} Application Editor used to control the LHS.

Calculation and data import into LHS and sample dilution scheme for binding affinity assay

Process samples were diluted in 96-well 2 mL deepwell plates starting in column 1 with a 1:10 dilution. Up to 4 serial 1:10 dilutions were carried out by transfer to columns 2 – 4 (see **Figure S2B**). Higher one-step dilution factors were avoided in all methods for accuracy reasons. In column 5, individual dilutions are performed for each sample to reach a common concentration for all samples (normalization step). In columns 6 to 9 all samples were diluted simultaneously with the 8-channel tool to levels around the dissociation constant K_D of FGF-2 to FGF receptor 2 which was found to be in the range of 1 to 4 nM.

A

Enter concentrations of samples

Location where to take sample for individual dilution

Copy volumes into csv-files

ID	Sample name	Conc. sample (mg/ml)	Conc. sample (nM)	Dil. factor for 20 nM	Dilution (logs)	Logs rounded down	dil. factor remaining	Vol dil. Sample (µl)	Vol. buffer (µl)	Final volume (20 nM)
1	1B1	0.121	7 034.9	351.7	2.5	2	3.5	170.6	429.4	600
2	1B2	0.239	13 895.3	694.8	2.8	2	6.9	86.4	513.6	600
3	1B3	0.688	40 000.0	2 000.0	3.3	3	2.0	300.0	300.0	600
4	1B4	1.946	113 139.5	5 657.0	3.8	3	5.7	106.1	493.9	600
5	1B5	5.471	318 081.4	15 904.1	4.2	4	1.6	377.3	222.7	600
6	1B6	13.631	792 500.0	39 625.0	4.6	4	4.0	151.4	448.6	600
7	1B7	22.889	1 330 755.8	66 537.8	4.8	4	6.7	90.2	509.8	600
8	1B8	23.413	1 361 220.9	68 061.0	4.8	4	6.8	88.2	511.8	600
9	1B9	17.386	1 010 814.0	50 540.7	4.7	4	5.1	118.7	481.3	600
10	1B10	8.948	520 232.6	26 011.6	4.4	4	2.6	230.7	369.3	600
11	1B11	3.544	206 046.5	10 302.3	4.0	4	1.0	582.4	17.6	600
12	1B12	1.093	63 546.5	3 177.3	3.5	3	3.2	188.8	411.2	600
13	1C1	0.419	24 360.5	1 218.0	3.1	3	1.2	492.6	107.4	600
14	1C2	0.277	16 104.7	805.2	2.9	2	8.1	74.5	525.5	600
15	1C3	0.188	10 930.2	546.5	2.7	2	5.5	109.8	490.2	600

B

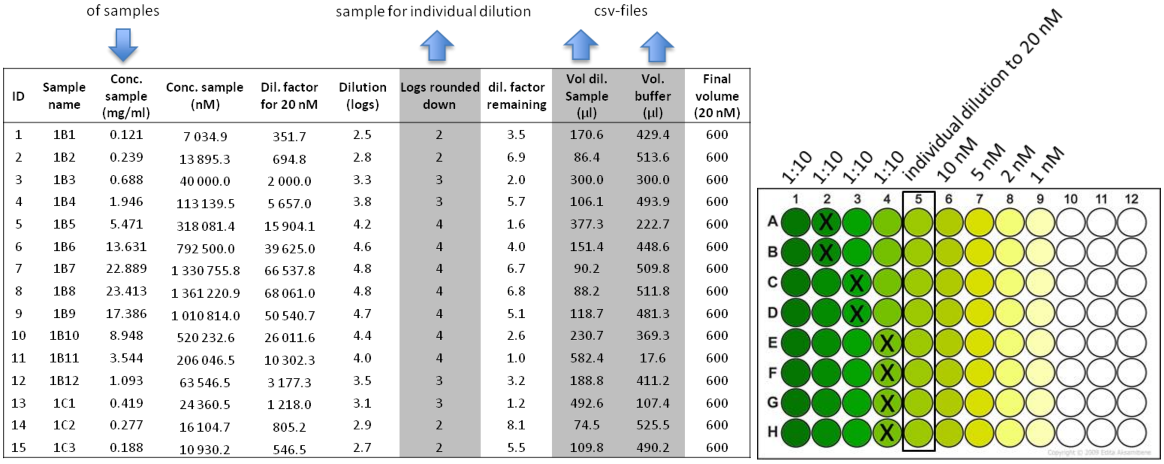

Figure S2: Sample preparation by semi-automated dilution for binding affinity measurement of FGF-2. (A) Calculation sheet to determine dilution steps. Csv-files are loaded into the LHS control software. (B) Scheme of dilution plate (96-well 2 mL deepwell plate). Every sample is diluted to 1, 2, 5, 10 and 20 nM.

Development of the plate wash procedure

A plate wash procedure for HCP ELISA was set-up on the LHS by sequentially dispensing and aspirating wash buffer. In order to remove as much of the wash solution out of the wells as possible in each step, the lowest tip position was determined at which none of the 8 tips touched the bottom of the wells. Keeping a distance to the bottom was important in order not to disturb the bound surface layer. This distance between well bottom and tip was eventually 0.8 mm. The remaining liquid volume after aspiration was 20 – 25 µL/well. In manual plate wash, wells are filled with multi-channel pipette and emptied by gravitational and mechanical force resulting in only the surface adsorbed liquid layer to remain in the wells.

Prior to the washing procedure the well were filled with a GFP solution in order to be able to rapidly and accurately determine the residual amount of protein. After each cycle of liquid aspiration and dispense of fresh wash buffer, the amount of remaining substance was determined by fluorescence measurement of GFP. The signal amplification factor (gain) was increased from 65 to 90 as the amount of substance decreased. Sequences with same number and one additional wash cycle were compared to manual plate wash for their efficiency to remove unbound substances (see Figure 3A). After manual wash the signal of GFP was 9% higher than the blank and in automated wash procedure the difference varied from 9 - 28%. (Figure 3B). The 16% difference compared to 9% was not considered as significant and therefore also included in further evaluations.

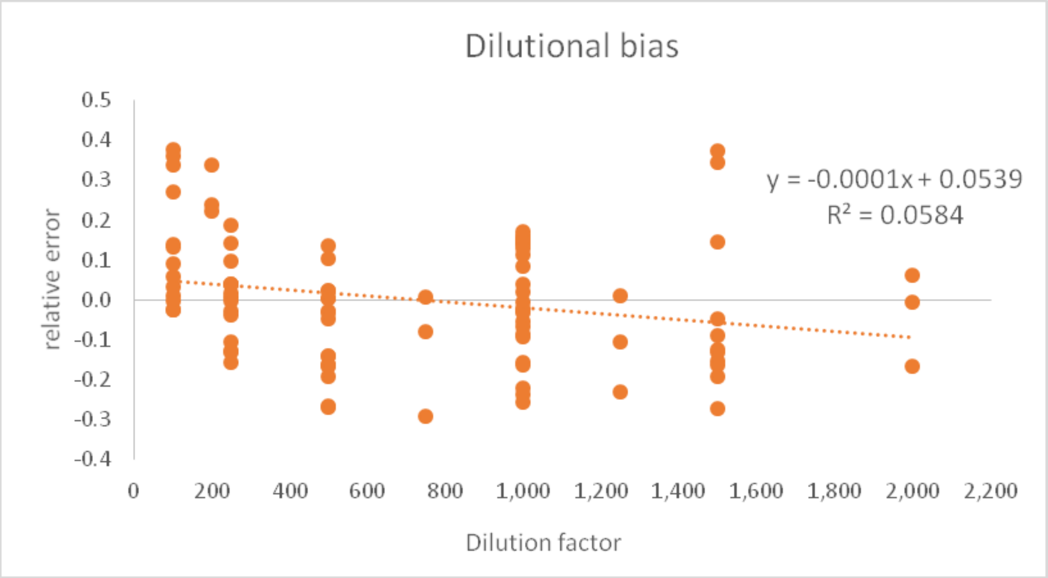

Figure S4: Dependence of results of DNA measurements on dilution factors (proportional bias).

Table S1: Details to Figure 5 on duration of assays in manual and semi-automated ways.

Analyte (Assay)		Same in both methods				Manual in semi-automated			Sample dilution [a]				Total	Working time saved
	Method	Steps	[min]	StdDev [min]	RSD	[min]	StdDev [min]	RSD	manual [min]	Automated [min]	StdDev [min]	RSD	[min]	[min]
Host Cell Proteins (ELISA)	manual	Preparations, plate coating and blocking, 4x plate wash, measuring absorbance, data evaluation, cleaning.	75.8	3.1	4.1%				43.3		0.577	1.3%	119.1	
	semi-automated					31.75	4.99	15.7%		62.7	1.50	2.4%	94.5	11.6
Endotoxins (Factor C)	manual	Preparations, mix reagent and add to samples, measure fluorescence, data evaluation, cleaning.	31.2	4.8	15.2%				55.5		4.5	8.1%	86.7	
	semi-automated					2.3	0.6	24.7%		53.7	0.1	0.2%	56.1	53.2
DNA (Picogreen)	manual		46.2	2.9	6.4%				18.7		1.53	8.2%	64.9	
	semi-automated					4.5	0.2	3.7%		25.6	1.1	4.4%	30.1	14.1
Binding affinity (Biacore)	manual	Calculation of dilutions, start SPR measuring, data evaluation, cleaning.	24	2.0	8%				53.7		2.3	4.3%	77.7	
	semi-automated					21.0	0.8	3.6%		27.6	0.3	1.1%	72.5	32.7
Total (ELISA, Endotoxin assay, Picogreen: 1x, Binding affinity: 2x) [b]									**224.9**	197.1				**144.3**

[a] For ELISA also staining and stop were done manually or automated.

[b] Binding affinity: 8 samples per assay.
